# Analysis and Reconstitution of the Menaquinone Biosynthesis Pathway in *Lactiplantibacillus plantarum* and *Lentilactibacillus buchneri*

**DOI:** 10.3390/microorganisms9071476

**Published:** 2021-07-09

**Authors:** Nisit Watthanasakphuban, Ludovika Jessica Virginia, Dietmar Haltrich, Clemens Peterbauer

**Affiliations:** 1 Department of Food Sciences and Technology, BOKU-University of Natural Resources and Life Sciences, Muthgasse 18, 1190 Vienna, Austria; faginsw@ku.ac.th (N.W.); jessica.virginia@boku.ac.at (L.J.V.); dietmar.haltrich@boku.ac.at (D.H.); 2 Department of Biotechnology, Faculty of Agro-Industry, Kasetsart University, Chatuchak, Bangkok 10900, Thailand

**Keywords:** menaquinone, demethlymenaquinone, respiration, lactic acid bacteria

## Abstract

In *Lactococcus lactis* and some other lactic acid bacteria, respiratory metabolism has been reported upon supplementation with only heme, leading to enhanced biomass formation, reduced acidification, resistance to oxygen, and improved long-term storage. Genes encoding a complete respiratory chain with all components were found in genomes of *L. lactis* and *Leuconostoc mesenteroides*, but menaquinone biosynthesis was found to be incomplete in Lactobacillaceae (except *L. mesenteroides*). *Lactiplantibacillus plantarum* has only two genes (*menA*, *menG*) encoding enzymes in the biosynthetic pathway (out of eight), and *Lentilactobacillus buchneri* has only four (*menA*, *menB*, *menE*, and *menG*). We constructed knock-out strains of *L. lactis* defective in *menA*, *menB*, *menE*, and *menG* (encoding the last steps in the pathway) and complemented these by expression of the extant genes from *Lactipl. plantarum* and *Lent. buchneri* to verify their functionality. Three of the *Lactipl. plantarum* biosynthesis genes, *lpmenA1*, *lpmenG1*, and *lpmenG2*, as well as *lbmenB* and *lbmenG* from *Lent. buchneri*, reconstituted menaquinone production and respiratory growth in the deficient *L. lactis* strains when supplemented with heme. We then reconstituted the incomplete menaquinone biosynthesis pathway in *Lactipl. plantarum* by expressing six genes from *L. lactis* homologous to the missing genes in a synthetic operon with two inducible promoters. Higher biomass formation was observed in *Lactipl. plantarum* carrying this operon, with an OD_600_ increase from 3.0 to 5.0 upon induction.

## 1. Introduction

Lactic acid bacteria (LAB) are non-respiring, fermentative anaerobic oxygen-tolerant bacteria and are typically cultivated under (micro)anaerobic conditions. In several LAB including *L. lactis* and *L. mesenteroides*, a respiration-like behavior was observed upon addition of heme to the medium [1], and actual respiration was shown in *L. lactis* [2] and *L. mesenteroides* [3,4], leading to stimulated aerobic growth, reduced acidification, improved growth efficiency, and stress resistance [2]. Furthermore, the addition of *Lacticaseibacillus casei* cultures grown under respiratory conditions led to improved organoleptic and nutritional properties of both cheddar cheese as well as sourdough [5,6]. The respiratory chain components of LAB are dehydrogenases, a heme-dependent cytochrome oxidase, and quinones as the electron shuttle [3,7,8]. For Gram-positive bacteria, vitamin K2 (menaquinone) is the sole quinone in the electron transport chain [9]. Menaquinone shuttle two electrons from electron donors and transfer them to the electron acceptor (heme) [9]. Since LAB do not produce heme [10] due to an incomplete heme biosynthesis pathway [11], it must be supplemented for all species. Supplementation of both heme and menaquinone leads to respiratory behavior in several additional species, indicating an incomplete respiratory chain lacking quinones [1,11].

In silico studies of LAB genomes revealed that the necessary genes for the formation of *bd*-type cytochrome oxidase (*cydABCD*) are widespread and are absent only in *Lactobacillus acidophilus*, *Lactobacillus delbrueckii*, and *Lactobacillus helveticus*, as well as in *Latilactobacillus curvatus* and *Latilactobacillus sakei* [1,3]. Genes that encode a complete menaquinone biosynthesis pathway are found in some *L. lactis* [12,13], but not Lactobacillaceae (with the exception of *L. mesenteroides*). The genome of *Lactipl. plantarum* WCFS1 contains only *menA* and *menG* genes, encoding the last two (of eight) pathway enzymes [14], and *Lent. buchneri* DSM 20057 contains *menE*, *menB*, *menA*, and *menG,* with the genes encoding the first four steps (*menFDHC*) in the pathway being absent. Both genomes contain the genes for the cytochrome oxidase (data from Joint Genome Institute, www.jgi.doe.gov, 9 May 2016, pathway analysis using the pathway maps of the Kyoto Encyclopedia of Genes and Genomes, www.genome.jp, 9 May 2016). 

In this study, we determined the functionality of the remaining menaquinone pathway genes in *Lactipl. plantarum* WCFS1 and *Lent. buchneri* DSM 20057 using classical methods of gene inactivation and genetic complementation for the investigation of a biosynthetic pathway and analyzed their growth behavior under aerobic conditions with heme supplementation. 

## 2. Materials and Methods

### 2.1. Bacterial Strains, Plasmids, Primers, and Culture Conditions

The relevant features of bacterial strains, plasmids, and primers used in this experiment are listed in Table 1, Table S1, and Table S2.

*L. lactis* subsp. *cremoris* NZ9000 was grown at 30 °C in M17 medium (Sigma-Aldrich, St. Louis, MO, USA) supplemented with 0.5% (*w/v*) glucose (GM17 broth) under anaerobic conditions (no agitation). *Lactipl. plantarum* WCFS1 and *Lent*. buchneri DSM 20057 were grown in MRS-medium (Roth) at 37 °C overnight under anaerobic conditions. *Escherichia coli* NEB5α and *Escherichia coli* JM101 were used as intermediate cloning host and were grown in LB broth at 37 °C with agitation at 150 rpm. Solid media contained 1.5% agar. Antibiotics were used for LAB at final concentrations of 10 µg/mL of chloramphenicol (cm) and 10 µg/mL or 30 µg/mL (for replica plating) of erythromycin (ery). For *E. coli*, the concentrations were 10 µg/mL (Cm) and 250 µg/mL (ery), respectively. 

Cultivation to assess aerobic growth was performed in baffled shake flasks filled to maximum 20% capacity with MRS broth at an agitation of 150 rpm. The growth medium was supplemented where indicated with menaquinone-4 (vitamin K2) (Sigma-Aldrich) at a final concentration of 20 ng/mL [1] or 1 µg/mL [6] and/or heme (Sigma-Aldrich) at a final concentration of 2 µg/mL. Menaquinone-4 was prepared as a stock solution at 2 mg/mL in ethanol, and heme was prepared as stock solution at 1 mg/mL in 0.05 M NaOH. Stock solutions were filtered using 0.2 µm sterile filters. As a control, an equivalent volume of ethanol or 0.05 M NaOH was added to non-supplemented media. Samples were taken after 24 h of growth for biomass (OD_600_) and acidification (pH) measurement. Growth and pH of induced and non-induced samples or with and without menaquinone supplementation samples were compared.

### 2.2. Knock-Out Vector Constructions

The construction of pNZ5319-∆*menA*, ∆*menB*, ∆*menE*, and ∆*menG* knock-out vectors was carried out by standard cloning procedures using restriction digestion and ligation [17]. The upstream and downstream fragments (1.0 kb each) of the target genes (*menA*, *menB*, *menE*, *menG*) were amplified from *L. lactis* NZ9000 chromosomal DNA using primer combinations (Table S2) and ligated into the compatible restriction sites of pNZ5319 upstream of *lox66* and downstream of *lox71*, respectively [17], and transformed into *E. coli* NEB5α competent cells. The knockout constructs were verified by colony PCR and sequencing.

### 2.3. L. lactis NZ9000 Gene Replacements

**Figure 1:**
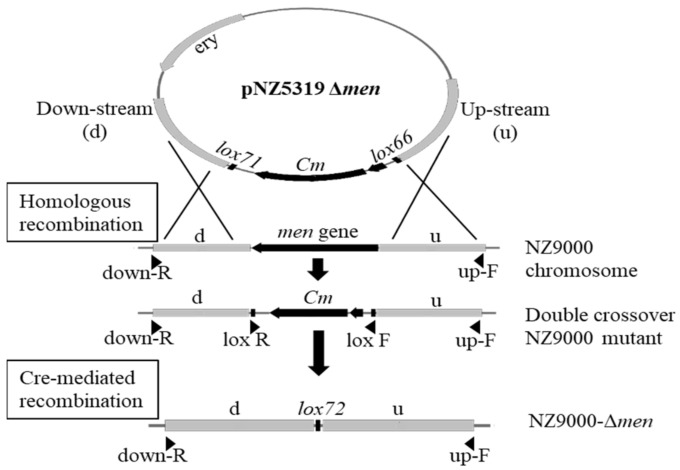
Construction of double-crossover men-gene replacement strains of *L. lactis* NZ9000 and subsequent Cre-lox-mediated selectable marker removal result in the *L. lactis* deletion strains (NZ9000-Δmen). After transformation with either of the pNZ5319 knockout constructs, double crossover events result in the replacement of the target gene (men: *menA*, *menB***,** *menE*, or *menG*) by the lox66-P32-cm-lox71 cassette. The cm-cassette is later removed by Cre-mediated recombination, resulting in non-functional lox72 sites.

The construction of the *menA*, *menB*, *menE*, and *menG* deletion strains was done using the Cre-*loxP* system (Figure 1). The pNZ5319-∆*menA*, ∆*menB*, ∆*menE*, or ∆*menG* plasmids were transformed into *L. lactis* NZ9000 competent cells [18,19] and selected for a chloramphenicol resistant and erythromycin sensitive phenotype, indicating double crossover at the homologous regions upstream and downstream of the *lox66*-P32-*cm*-*lox71* cassette and loss of the plasmid backbone carrying the erythromycin resistance gene. The correct integration of the *lox66*-P32-*cm*-*lox71* cassette and the absence of the coding sequences of *menA*, *menB*, *menE*, or *menG* and *ery* were confirmed with primers, as listed in Table S2. 

The antibiotic selection marker cassette (*lox66*-P32-*cm*-*lox71*) in the chromosomes of the knockout strains of *L. lactis* NZ9000 was excised using a transient Cre recombinase expression plasmid (pNZ5348) [17]. A total of 4–6 µg of pNZ5348 was transformed into the double crossover strains, and ery^r^ colonies were picked and tested for Cre-mediated recombination, using primers spanning from upstream to downstream of the recombination locus (Table S2, Figure 1). The pNZ5348 plasmid was cured from the strains by growth without erythromycin selection pressure for about 10 generations, then 100–300 colonies were picked into microtiter plates with GM17 and tested for an ery^s^ phenotype, indicating loss of the plasmid pNZ5348 [20]. Knockout genotypes were confirmed by sequencing (Microsynth, Balgach, Switzerland) using primers upstream and downstream of the respective loci as mentioned above. 

### 2.4. Construction of Expression Vectors for Menaquinone Biosynthesis Genes 

The nucleotide sequences coding for *menA1, menA2, menA3, menG1*, and *menG2* genes from *Lactipl*. *plantarum* WCFS1 and *menA*, *menB*, *menE*, and *menG* genes from *Lent*. buchneri DSM 20057 were amplified using appropriate primers (Table S3) and ligated into the nisin-inducible expression vector pNZ8150. Ligation mixtures were transformed into *E. coli* JM101 (*recA*+) competent cells and verified by sequencing using primer pnz8150F and pnz8150R (Table S3). Plasmids for the expression of the homologous *L. lactis* NZ9000 genes *menA*, *menB*, *menE*, and *menG* as controls were constructed analogously. 

The *L. lactis* NZ9000 menaquinone biosynthesis-deficient strains (∆*menA*, ∆*menB*, ∆*menE*, or ∆*menG*) (Table 1) were transformed with the appropriate expression vector for the respective menaquinone biosynthesis gene (Table S4).

### 2.5. Complementation of Deleted Menaquinone Biosynthesis Genes and Menaquinone Extraction

The resulting strains (Table S5) were investigated for menaquinone formation. The transformant strains were cultivated following the manufacturer’s protocol for the NICE Expression System for *L. lactis*. Overnight pre-cultures were diluted 1:100 into 500 mL fresh GM17 broth, grown to an optical density OD_600_ = 0.4, induced with 1 ng/mL nisin, and incubated further at 30 °C for 24 h [1]. Cells were harvested by centrifugation at 5000× g for 15 min, washed twice with 1 volume potassium phosphate buffer (PPB; pH 6.0), and stored at −20 °C until used for analysis.

Menaquinone extraction was performed in dark containers to avoid light-dependent degradation. A total of 1 g of cell biomass was transferred to an amber glass bottle and suspended into 1.5 mL PPB, 6.0 mL of methanol/acetone (1:1 (*v/v*)) [21] was added, and the suspension was vigorously agitated on an orbital shaker at 250 rpm for 30 min at room temperature. Then, 2 mL of petroleum ether was added, and the mixture was again agitated for 10 min at 250 rpm. Following phase separation by 1 min centrifugation (2800× *g* at 15 °C), the upper layer was removed. The lower layer was re-extracted with 2 mL petroleum ether, the upper layer was again removed, and the two combined lower fractions were evaporated under nitrogen gas at room temperature. The dried residues containing menaquinone were dissolved in 10 µL ethanol [1,22] for analysis.

### 2.6. Thin-Layer Chromatography (TLC)

The obtained extracts were analyzed using TLC, as described previously [23], with some modifications on Merck Kieselgel 60 F254 plates (Merck, Darmstadt, Germany). Hexane-diethyl ether (85:15) was used as the solvent system in the saturated chamber. The plates were dried and examined under UV light (254–365 nm), and purified menaquinone-4 (Sigma-Aldrich) was used as standard.

### 2.7. Growth Profile Analysis

Parent strains with disrupted biosynthesis genes were compared with transformant strains expressing the respective complementing gene from *Lactipl. plantarum* WCFS1 and *Lent*. buchneri DSM 20057 or *L. lactis* NZ9000 as control. Cultivation and induction were performed in aerobic condition as described above, and samples were taken for pH measurement and OD_600_ in three-hour intervals.

### 2.8. Construction of Expression Vectors for Menaquinone Biosynthesis

For expression of menaquinone biosynthesis genes in *Lactipl. plantarum* WCFS1, the vector pSIP409 was used. An internal *Bsa*I site was eliminated using site-directed mutagenesis [24] with the primers pSIP *Bsa*I KO F and pSIP *Bsa*I KO R (Table S3). The vector backbone was amplified from the modified pSIP409 (pSIP409∆*Bsa*I) with the primers pSIP409 F and pSIP409 R with *Bsa*I restriction sites and complementary overhangs (Table S3). Menaquinone biosynthesis genes were amplified from the genome of *L. lactis* NZ9000 with primers as listed in Table S3, containing 5′ flanking bases, a *Bsa*I restriction site, and a four-base overhang. A second promoter (P_sppQ__2) fragment was amplified from pSIP409 as above with primers PsppQ F and PsppQ R. 

Golden Gate Assembly was used to assemble the multiple DNA fragments and vector backbones in the form of synthetic operons encoding all six genes, with and without a second inducible promoter (PsppQ_2) inserted between the third and fourth genes. Oligonucleotide primer sequences are in Supplementary Material Table S6.

The assembly reactions were set up following the instruction manual of NEB Golden Gate Assembly Kit (New England BioLabs, Ipswich, MA, USA) and resulting in pSIP_lpMK_2 and pSIP_lpMK_1 (Table 2). The constructs with the expected size were confirmed by sequencing (Microsynth, Balgach, Switzerland) before being transformed into *Lactipl*. *plantarum*-competent cells.

### 2.9. Analysis of Aerobic Phenotype of Lactipl. plantarum variants 

The growth behavior of *Lactobacillus* variants and their wild type (control) was investigated under aerobic conditions, and overnight cultures were transferred into 100 mL MRS broth containing 5 µg/mL ery supplemented with 2 µg/mL heme in 500 mL baffled flasks, and were incubated at 37 °C, 150 rpm agitation speed. For the strains carrying an inducible plasmid, 25 ng/mL of SppIP was added at OD_600_ = 0.3, and sterile water was added to the controls and strains carrying the plasmid with the constitutive promoters. The OD_600_ and pH of the supernatant were measured at 0 h and 24 h incubation. Growth and pH of induced and non-induced clones were compared.

## 3. Results

### 3.1. Construction of Menaquinone Knockout Vectors 

The knockout vectors were constructed using pNZ5319, which contains the *ery* antibiotic resistance marker as well as the *lox66*-P32-*cm*-*lox71* cassette. This cassette was flanked with upstream and downstream sequences of the genes encoding the target MK biosynthesis genes (*menA*, *menB*, *menE*, or *menG*, respectively) to provide homologous recombination sites. Cm^r^ colonies were verified for correct construction before transformation into *L. lactis*. Around 80% of the clones showed double crossover integration with replacement of the biosynthesis genes. Correct integration of the *lox66*-P32-*cm*-*lox71* cassette and absence of *menA*, *menB, menG*, and *menE* were confirmed by PCR as shown in Figure S1: *menA*, *menB, menE*, and *menG* (906, 843, 1356, and 741 bp, respectively) were replaced by the 1038 bp of *lox66*-P32-*cm*-*lox71* cassette. Representative strains were verified by sequencing before the next step was performed.

Cre-mediated recombination was performed for the removal of P32 and the antibiotic resistance gene (*cm*) from the *L. lactis* genome using a plasmid carrying the Cre recombinase gene (pNZ5348) [17]. Ery^r^ colonies containing pNZ5348 were checked for *lox66*/*lox71* mediated recombination and excision of the marker cassette by a cm^s^ phenotype and by verifying the loss of the 1038 bp-cassette (A-3, B-3, E-3, and G-3) (Figure S1). The presence of the non-functional *lox72* site [17] resulting from *lox66*/*lox71* recombination was confirmed by sequencing. The transient Cre expression vector (pNZ5348) was then cured through cultivation for 10 generations without erythromycin. About 5% showed the ery^s^ phenotype under nonselective conditions. The *L. lactis* ∆*menA*, *L. lactis* ∆*menB*, *L. lactis* ∆*menE*, and *L. lactis* ∆*menG* knockout strains were used for the functional verification of the extant menaquinone biosynthesis genes from *Lactipl*. *plantarum* WCFS1 and *Lent*. buchneri DSM 20057.

### 3.2. Expression of Menaquinone Genes from Lactipl. plantarum WCFS1 

The functionality of the menaquinone biosynthesis genes from *Lactipl*. *plantarum* WCFS1 and *Lent*. buchneri DSM 20057 was tested by analysis of menaquinone production and of growth profiles. The study of menaquinone production was conducted under anaerobic conditions, which resulted in a twofold yield of menaquinone production in *L. lactis* MG 1363 [1].

**Figure 2:**
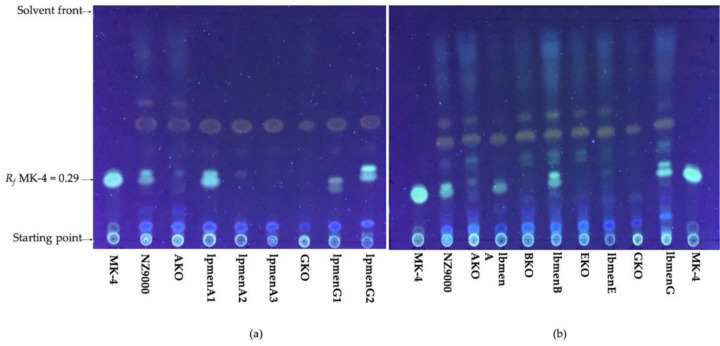
Menaquinone detection on TLC of the menaquinone extracts from *L. lactis* NZ9000; its deficient strains ∆*menA* (AKO), ∆*menB* (BKO), ∆*menE* (EKO), and ∆*menG* (GKO); and the engineered strains with complementing biosynthesis genes from (**a**) *Lactipl*. *plantarum* WCFS1 (*lpmenA1*, *lpmenA2*, *lpmenA3*, *lpmenG1*, *lpmenG2*) and (**b**) *Lent*. *buchneri* DSM 20057 (*lbmenA*, *lbmenB*, *lbmenE*, *lbmenG*). Menaquinone with four isoprenoid residues was used as standard (MK-4).

Menaquinone was extracted from all strains using solvent extraction and detected in TLC using menaquinone with four isoprenoid residues (MK-4) and a retardation factor (*R_f_*) of 0.29 as standard. Six of nine engineered strains, namely, those harboring *lpmenA1*, *lpmenG1*, *lpmenG2*, *lbmenA*, *lbmenB*, and *lbmenG* as well as *L. lactis* NZ9000 wild type (WT) showed bands representing menaquinone (Figure 2). The menaquinone band was absent in the extracts from all knockout strains (*L. lactis* ∆*menA*, *L. lactis* ∆*menB*, *L. lactis* ∆*menE*, and *L. lactis* ∆*menG*). This confirms the successful abolishment of menaquinone biosynthesis by knocking out *menA*, *menB*, *menE*, or *menG,* as well the absence in *L. lactis* of orthologs of the *men* genes or of an alternative menaquinone biosynthetic pathway, as was reported for *Helicobacter* and *Chlamydia* strains [26]. 

The menaquinone extract from the WT strain appeared as two nearly comigrating bands with *R_f_* values of 0.29 and 0.32 on TLC under UV detection. The same profile was detected in the menaquinone extracts from the strains carrying *lpmenA1*, *lpmenG1*, and *lpmenG2*; the extant menaquinone biosynthesis genes from *Lactipl*. *plantarum* WCFS1 (Figure 2a); and the strains carrying the biosynthesis genes *lbmenA*, *lbmenB*, and *lbmenG* from *Lent*. *buchneri* DSM 20057 (Figure 2b). The different *R_f_* values for MK-4 (*R_f_* = 0.29) and the higher band (*R_f_* = 0.32) could be due to the production of menaquinone in varying side chain lengths (isoprenoid residues) from MK-2 to MK-10 of *L. lactis* [1,27,28] or a detection artefact. In anaerobic conditions, *L. lactis* NZ9000 produces two dominant menaquinone variants (MK-3 and MK-9) [1], which are obviously seen in TLC as multiple menaquinone spots in the extracted samples (Figure 2). The *R_f_* of menaquinone homologues are readily separated in TLC [29,30,31] if the difference amounts to more than three isoprene units [30]. 

In contrast, no menaquinone could be detected in extracts of the strains expressing *lpmenA2*, *lpmenA3*, and *lbmenE* (Figure 2). 

The menaquinone extracts from *L. lactis* NZ9000 and its menaquinone deficient strains ∆*menB* (BKO), ∆*menE* (EKO), and ∆*menG* (GKO) were checked for MK-6, which was found to be absent in these strains, but appeared in the WT sample (Figure S2), confirming the successful knockout of menaquinone biosynthesis in these strains.

### 3.3. Growth Profile Analysis

**Figure 3:**
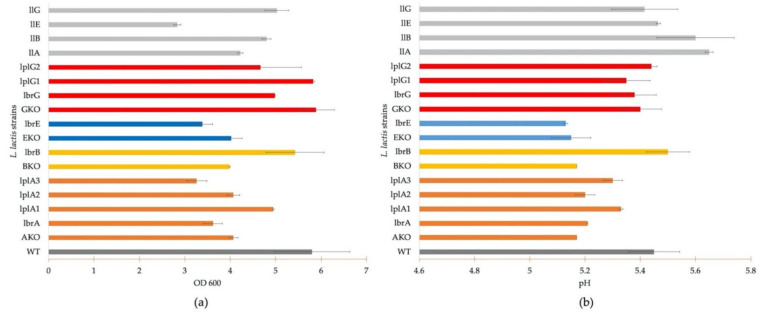
Growth (**a**) and pH profile (at 24 h incubation) (**b**) of *L. lactis* NZ9000 (WT), its menaquinone deficient strains (∆*menA*, ∆*menB*, ∆*menE*, ∆*menG*), *L. lactis* engineered strains carrying (*lbmenA*, *lpmenA1*, *lpmenA2*, *lpmenA3*, *lbmenB*, *lbmenE*, *lbmenG*, *lpmenG1*, *lpmenG2*), and engineered deficient strains complemented by homologous genes (*llmenA*, *llmenB*, *llmenE*, *llmenG*). A total of 300 mL of GM17 medium supplemented with heme (2 µg/mL) was used, and 1 ng/mL of nisin was added as inducer at OD_600_ = 0.4. Growth curves are presented in Supplementary Materials Figures S3–S5.

The growth profiles of *L. lactis* NZ9000 (WT) and ∆*menG* showed a high cell density (OD_600_ about 5.8), whereas the *L. lactis menA-*, *menB-*, and *menE*-deficient strains showed an OD_600_ of about 4.0 under aerobic cultivation conditions with heme supplementation (Figure 3a). Interestingly, among the knockout strains, *L. lactis* ∆*menG* showed a cell density that was comparable to the wild type (Figure 3a). The *menG* gene encodes demethylmenaquinone methyltransferase (MenG), which adds a methyl group to the aromatic ring of demethylmenaquinone (DMK) and is the final step of menaquinone biosynthesis [32,33].

The functional verification of the *menA* genes from *Lactipl*. *plantarum* WCFS1 and *Lent*. buchneri DSM 20057 is shown in Figure 3a. An increased cell density (OD_600_) was detected in the strain expressing the *menA1* gene from *Lactipl*. *plantarum* WCFS1 with a maximum OD_600_ of 4.9. *L. lactis* ∆*menA*, and the strain expressing *lpmenA2* showed a maximum OD_600_ of only 4.0, and lower growth was detected in the strains expressing *lbmenA* and *lpmenA3* with OD_600_ of 3.6 and 3.2, respectively (Figure 3a). The complementation of *L. lactis* ∆*menB* by expression of *lbmenB* from *Lent*. *buchneri* DSM 20057 exhibited a maximum OD_600_ of 5.0 at 24 h incubation compared to an OD_600_ of 4.0 in *L. lactis* ∆*menB*. In contrast, *L. lactis* ∆*menE* reached an OD_600_ = 4.0, whereas the strain expressing *lbmenE* showed an OD_600_ of 3.4 after 24 h incubation (Figure 3a). 

The comparison of the growth profile between *L. lactis* ∆*menG* and its derivative strains expressing *lbmenG*, *lpmenG1*, or *lpmenG2* gene showed a high growth phenotype during 24 h incubation for all strains. The highest OD_600_ at 24 h was observed in *L. lactis* ∆*menG* and the strain harboring *lpmenG1,* followed by the strains carrying *lpmenG2* and *lbmenG*, with maximum OD_600_ values of 5.8, 5.8, 5.3, and 5.1, respectively.

The respiratory metabolism in *L. lactis* not only promotes biomass formation but also results in decreased lactic acid production [1]; thus, the pH of the cultures was monitored. The reduction of pH during growth of *L. lactis* NZ9000 and its *men*-deficient derivative strains is shown in Figure 3b. A higher pH of about 5.4 was observed in *L. lactis* NZ9000 and *L. lactis* ∆*menG* strains after 24 h. A lower pH was observed in the ∆*menA,* ∆*menB*, and ∆*menE* strains. *L. lactis* ∆*menG*, where only the last gene in the menaquinone biosynthesis pathway is knocked out, has uninhibited high growth and reduced acid production under aerobic conditions (Figure 3).

The pH monitoring during growth of *L. lactis* ∆*menA* and the complementation strains also verifies functionality of *lpmenA1* from *Lactipl*. *plantarum* WCFS1. *L. lactis lpmenA3* showed a pH as high as *L. lactis lpmenA1* (5.3) but at lower growth. *L. lactis lbmenA* and *lpmenA2* showed a low pH comparable to the deficient strain *L. lactis* ∆*menA*. *L. lactis lbmenA* showed a faint menaquinone band on TLC (Figure 2), but growth and pH profile were not restored to (near) WT levels. 

The monitored pH curves show that *lbmenB* restored the respective growth phenotype, with a pH at 24 h of 5.6. The gene *lbmenE* was not able to restore menaquinone production, as shown by growth (Figure 3) and menaquinone detection on TLC (Figure 2). 

The knockout mutations were also complemented by expression of the homologous menaquinone biosynthesis genes (*llmenA, llmenB, llmenE, llmenG*) from *L. lactis* NZ9000 in order to determine potential differences between homologous and heterologous genes/enzymes as well as the metabolic load of plasmid maintenance to cell growth. The knockout strains expressing *llmenA* and *llmenB* showed higher growth than the deficient strains at an OD_600_ of 4.2 and 4.8, respectively. *L. lactis llmenG* exhibited slightly lower growth (OD_600_ = 5.0) than *L. lactis* ∆*menG* (OD_600_ = 5.8), but the cell growth was comparable to other *menG* expression strains (Figure 3). Lower biomass formation was observed in the *L. lactis llmenE* with the OD_600_ of 2.8, similar to *L. lactis* *lbmenE*, and both were lower than *L. lactis* ∆*menE*.

The pH profile of all strains expressing homologous genes showed higher pH than the respective deficient strains ranging from pH 5.4 to 5.6 (Figure 3b), except *L. lactis* *lbmenE*, where a higher final pH corresponded to low growth.

### 3.4. Respiratory Phenotype of Lent. buchneri DSM 20057 and Lactipl. plantarum WCFS1 and Variant Strains

**Figure 4:**
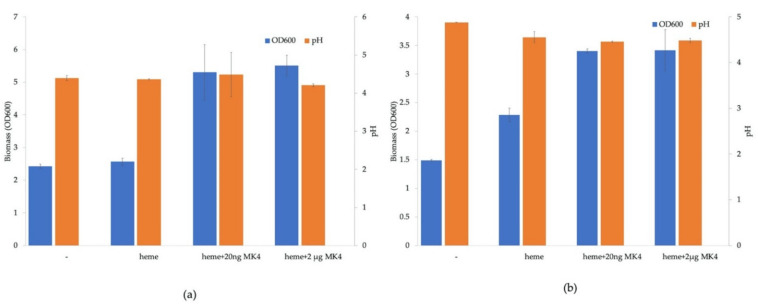
*Lactipl*. *plantarum* WCFS1 (**a**) and *Lent*. *buchneri* DSM 20057 (**b**) were grown aerobically for 24 h at 37 °C in MRS medium without heme and menaquinone supplementation (-), with only heme supplementation (heme), and with heme and 20 ng/mL or 2 µg/mL menaquinone supplementation (heme + MK4). The blue bars represent the biomass formation (OD_600_), and the orange bars represent the final pH.

Both *Lent*. *buchneri* and *Lactipl*. *plantarum* were cultivated under aerobic conditions with supplementation of both heme and menaquinone (Figure 4). In *Lactipl*. *plantarum* WCFS1, OD_600_ increased from 2.4 without heme and menaquinone supplementation to 4.5 (Figure 4a). In *Lent*. *buchneri* DSM 20057, OD_600_ increased from 1.5 without supplementation to 3.4 when supplemented with heme and menaquinone (Figure 4b). The final pH after 24 h cultivation of *Lactipl*. *plantarum* was about 4.3 in all samples, with and without supplementation (Figure 4a). The final pH of *Lent*. *buchneri* DSM 20057 without exogenous heme and menaquinone was 4.9; with supplementation, the final pH was slightly lower at 4.5 (Figure 4b). Menaquinone was supplied at 20 ng/mL and 2 µg/mL, but no concentration-dependent differences were observed in biomass formation or in final pH (Figure 4a,b).

**Figure 5:**
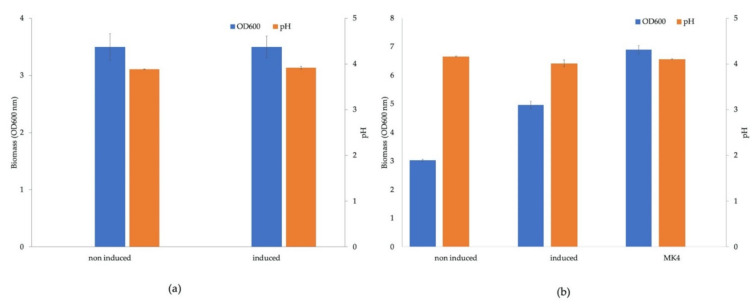
Cultivation of *Lactipl. plantarum* carrying the plasmids pSIP_lpMK_1 (**a**) and pSIP_lpMK_2 (**b**). The engineered strain was grown aerobically for 24 h at 37 °C in MRS medium with IP (induced) and without IP (non-induced) as the inducer. The blue bars represent the biomass formation (OD_600_), and the orange bars represent the final pH.

*Lactipl*. *plantarum* was transformed with an expression vector (pSIP_lpMK_1*)*, which carried the six menaquinone biosynthesis genes controlled by one promoter. The resulting strain showed the same growth behavior under induced and non-induced conditions (Figure 5a), and identical levels of pH were measured in both samples. The expression vector pSIP_lpMK_2 carries the six menaquinone biosynthesis genes with an additional inducible promoter between the third and fourth gene. A *Lactipl*. *plantarum* strain carrying this construct showed higher biomass formation under aerobic conditions with heme supplementation upon induction of the biosynthesis genes. The OD_600_ of the sample without induction after 24 h cultivation was about 3.0, and the induced sample showed an OD_600_ of about 5.0 (Figure 5b). The control sample, where heme and menaquinone were added, gave an OD_600_ of 7.0. The final pH measurement revealed that all three samples had a pH around 4.1 (Figure 5b).

## 4. Discussion

To verify the functionality of the existing menaquinone biosynthesis genes from *Lactipl*. *plantarum* WCFS1 *(menA*, *menG*) and *Lent*. *buchneri* DSM 20057 (*menA*, *menB*, *menE*, *menG*), we constructed respective knock-out strains in *L. lactis* (which has a complete set of menaquinone biosynthesis genes) using the Cre-*loxP* system with selection marker removal by Cre recombinase [17]. Deletion of *menA*, *menB*, and *menE* had the expected effect on the growth behavior of *L. lactis* NZ9000 due to incomplete menaquinone biosynthesis, i.e., higher biomass formation and reduced medium acidification associated with respiratory metabolism in aerobic cultivation was abolished. An exception was *L. lactis* NZ9000 ∆*menG*, which showed cell densities and medium acidification comparable to the wild type *L. lactis* NZ9000 (Figure 3a). *MenG* encodes demethylmenaquinone methyltransferase, the enzyme catalyzing the final step of menaquinone biosynthesis: conversion of DMK to MK by addition of a methyl group to the aromatic ring of DMK [28,29]. *E. faecalis* only synthesizes DMK but has also shown respiratory growth upon medium supplementation with heme [33,34,35,36], and a *Staphylococcus aureus* ∆*menG* strain exhibited functional electron transport using DMK [27]. These observations suggest that DMK is functional as electron shuttle in the electron transport chain.

Expression of *Lactipl*. *plantarum menA1* led to restoration of menaquinone biosynthesis and a growth behavior comparable to the wild type upon aerobic cultivation, i.e., higher biomass formation and reduced acidification. From this, we conclude that the extant *menA1* gene still encodes a fully functional enzyme in *Lactipl*. *plantarum*. Expression of *menA2* or *menA3* from *Lactipl*. *plantarum* in *L. lactis* Δ*menA* did not fully restore the growth phenotype or menaquinone production as analyzed by TLC. Thus, the respective genes, while appearing to be intact in the genome (i.e., there are no obvious larger deletions or insertions or mutations causing a frameshift), no longer encode functional enzymes due to the accumulation of non-functionalizing mutations. The strain expressing *menA* from *Lent*. *buchneri* displayed menaquinone production in TLC, but markedly weaker than in the other, successfully complemented strains, and a respiration-associated growth phenotype could not be restored. The most likely explanation is that these genes have, in the absence of selective pressure on functionality, accumulated mutations that have not entirely abolished its function but compromised the catalytic activity of the enzyme, leading to production levels in the complementation strain that are, albeit detectable, insufficient for a functional electron transport chain. 

*MenE* expression produced results (in all tests) almost undistinguishable from the knock-out strain, suggesting that the gene no longer encodes an intact enzyme. In this case, however, complementation with the homologous *llmenE* was equally unsuccessful. Closer examination revealed that the design of the knock-out cassette is probably responsible for this: *menE* is overlapping the immediately following *menC* by four nucleotides, i.e., TG in the start codon and A in the second codon (ATA, Ile) of the *menC* coding region constitute the *menE* stop codon TGA. This was taken into account, and the disruption cassette was constructed using the intact *menC* coding sequence as downstream homologous element for the integration of the marker cassette. Thus, integration of the construct via double crossover also removes the *menC* ribosome binding site (RBS). Cre-recombinase-mediated excision of the cassette via the *lox66*/*lox71* sites should move the *menE* RBS to a position upstream of the *menC* start codon; however, the remaining *lox72* site increases the distance between RBS and start codon by more than 30 nts. This could affect translation of *menC* and lead to lowered enzyme titers of active MenC. If that is indeed the case, complementation of the *menE* knock-out will be unsuccessful because of insufficient MenC-activity, even if the complementing *menE* gene is functional. We did not determine MenC activity or quantify MenC in the knock-out or the complemented strain, and the complementation experiment with *Lent*. *buchneri menE* is therefore inconclusive. On the basis of our initial interpretation, that *Lent*. *buchneri* *menE* no longer encodes a functional enzyme, *lbmenE* was included in the expression constructs for pathway restoration. 

Deletion of *L. lactis menG* did not give a conclusive growth phenotype, as described above, and thus complementation by any of the genes encoding the demethylmenaquinone methyltransferase*, menG1* and *menG2* from *Lactipl*. *plantarum*, and *menG* from *Lent*. *buchneri* cannot be detected. TLC analysis of the respective strains, however, clearly shows restored menaquinone production in all the strains expressing either one of these three genes, indicating that all three genes encode functional enzymes. 

TLC analysis of extracts of the various *L. lactis* strains generally gave spots with two slightly different *R_f_* values near the position of MK-4. Menaquinone occurs in side chain lengths (number of isoprenoid residues) from MK-2 to MK-10 in *L. lactis* [1,27,28]. Under anaerobic conditions, two dominant menaquinones, MK-3 and MK-9, are detected [1], which can be seen in TLC (Figure 2). Menaquinones are readily separated in TLC [29,30,31] if the difference is more than three isoprene units [30]. 

In reports on *L. lactis*, respiratory metabolism led to both higher biomass formation as well as lower acidification of the growth medium [1]. Under fermentation conditions, lactate dehydrogenase (LDH) produces lactate from pyruvate, concomitantly regenerating NAD^+^ from NADH. Under aerobic conditions and heme supplementation, the encoding gene *ldh* was downregulated, and the gene encoding pyruvate dehydogenase (*pdh*) was upregulated [37]. This results in formation of acetate and acetoin production at the expense of lactate [7,38], leading to less pronounced acidification and higher final pH, while maintaining NADH consumption/NAD^+^ regeneration through NADH oxidase [39]. In our results, we observed higher biomass formation upon respiratory metabolism, but not a lower final pH, in both wild type strains (*Lactipl*. *plantarum* WCFS1 and *Lent*. buchneri DSM 20057), as well as the engineered *Lactipl*. *plantarum* strain. A possible explanation is that acetoin production is absent in *Lactipl*. *plantarum*, leading to (mostly) acetate production instead of lactate, which may result in the same final pH. Besides that, doubt has been cast onto the rerouting of metabolism via PDH due to proteomic studies [40]. Investigation of the transcriptomic response to aerobic conditions, heme supplementation, and menaquinone biosynthesis will be part of future work. 

Interestingly, supplementation with only heme led to a higher biomass formation in *Lent. buchneri* DSM 20057 in aerobic cultivation (Figure 4b), but lower than upon supplementation with heme and menaquinone. The genes for oxidases (pyruvate oxidase, lactate oxidase) are upregulated in aerobic conditions together with other genes [41]. This can lead to an accumulation of hydrogen peroxide and free radicals, resulting in oxidative damage to cellular components [42]. Supplementation of heme under aerobic conditions induces the production of heme-dependent catalase and thus to degradation of hydrogen peroxide [3,42,43] and alleviation of oxidative stress. This was also observed in *L. rhamnosus* in the presence of heme after expression of a gene encoding heme-dependent catalase (*KatA*) [44]. However, this effect was not observed under aerobic conditions in *Lactipl. plantarum* WCFS1 (Figure 4a), although increased expression of catalase under aerobiosis was shown in *Lactipl. plantarum* as well [45]. *Lactipl. plantarum* was shown to produce a Mn-dependent (pseudo)catalase [42,43,46] that decomposes hydrogen peroxide under aerobic conditions without requiring heme, as well as another, Mn(II)-based and heme-independent stress resistance mechanism to detoxify superoxide [47], and it is not clear to what extent these different mechanisms contribute to oxidative stress response.

In a first trial at pathway reconstruction, we assembled the six menaquinone biosynthesis genes (each with its own RBS) into a polycistronic operon under control of a single promoter. On the basis of the observed growth behavior, we found that this did not result in respiratory metabolism. Reports in literature suggest that transcription of mRNAs beyond a certain length leads to a low ratio of full-length RNAs and consequently low yields of the protein(s) encoded by the gene(s) furthest downstream from the promoter [48]. A limit of four genes (of average length) transcribed from one promoter is suggested [49]. We conclude that the last or the two last genes in the construct (*menC* and *menE*) are transcribed only at low levels (i.e., that full-length mRNAs containing these two coding regions are rare), resulting in levels of functional enzyme that are too low to produce sufficient amounts of menaquinone. This can be remedied by positioning of a strong promoter upstream of every gene [50]. Since the required construct consists of six genes, assembly of these genes with six promoters was considered cumbersome and inefficient, whether by restriction and ligation or by Golden Gate Assembly. We therefore decided on an expression vector with two operons of three genes, each controlled by the same inducible promoter. The genes were placed in the same order as in the genome of *L. lactis* NZ9000, with *menF*, *mend*, and *menH* controlled by the first promoter, followed by the second promoter controlling the expression of *menB*, *menE*, and *menC*. Strains transformed with this construct could, upon induction of the two promoters controlling the menaquinone biosynthesis genes, produce higher biomass (Figure 5b), indicating a successful restoration of menaquinone biosynthesis and a complete respiratory chain.

## Figures and Tables

**Table 1:** Bacterial strains used in the biosynthesis gene knockout study.

Strains	Relevant Features	References
*Escherichia coli*		
NEB5α	Cloning host	NEB
JM101	Cloning host *recA*^+^	[15]
*Lactococcus lactis* (ll)		
NZ9000	MG1363 *pepN*::*nisRK*, cloning host	[16]
∆*menA*	NZ9000 *menA* deficient by *lox72* replacement	This study
∆*menB*	NZ9000 *menB* deficient by *lox72* replacement	This study
∆*menE*	NZ9000 *menE* deficient by *lox72* replacement	This study
∆*menG*	NZ9000 *menG* deficient by *lox72* replacement	This study

**Table 2:** Plasmids used and menaquinone expression plasmid constructs for two Lactobacillus strains.

Plasmids	Relevant Features	References
pSIP409gusA	*spp*-based expression vector, PSIP401 derivative, *gusA* controlled by P*_sppQ_*(P_orfX_), *sppKR* expression driven by *eryB* read through	[25]
pSIP409∆*Bsa*I	ery, PSIP401 derivative with removal of *Bsa*I restriction site	This study
pSIP_lpMK_2	ery, PSIP401∆*Bsa*I derivative, menaquinone biosynthesis genes for *Lactipl. plantarum* controlled by two P*_sppQ_*	This study
pSIP_lpMK_1	ery, PSIP401∆*Bsa*I derivative, menaquinone biosynthesis genes for *Lactipl*. *plantarum* controlled by one P*_sppQ_*	This study

## Supplementary Materials

The following are available online at https://www.mdpi.com/article/10.3390/microorganisms9071476/s1, Figure S1: Diagnostic PCR of L. lactis NZ9000 menA (A-1), its gene replacement mutant menA:: lox66-P32-cm-lox71 (A-2), ∆menA (A-3); menB (B-1), menB:: lox66-P32-cm-lox71 (A-2), ∆menB (B-3); menE (E-1), menE:: lox66-P32-cm-lox71 (E-2), ∆menE (E-3); menG (G-1), menG:: lox66-P32-cm-lox71 (A-2), ∆menG (G-3); M is a 2 log DNA ladder, Figure S2: Base peak chromatogram of menaquinone extracts from L. lactis NZ9000 (WT) and its menaquinone deficient strains ∆menB (BKO), ∆menE (EKO), ∆menG (GKO) for MK-6, Figure S3: Growth profile analysis of (a) L. lactis NZ9000 (WT) and the knockout strains (∆menA, ∆menB, ∆menE, ∆menG), (b) L. lactis ∆menA and its engineered derivatives carrying lbmenA, lpmenA1, lpmenA2 and lpmenA3), (c) L. lactis ∆menB, L. lactis ∆menE and the engineered derivatives (carrying lbmenB, lbmenE) and (d) L. lactis ∆menG and its engineered derivatives carrying lbmenG, lpmenG1 and lpmenG2. 300 mL of GM17 medium supplemented with heme (2 µg/mL) were used, 1 ng/mL of nisin was added as an inducer at OD600 = 0.4, Figure S4: pH profile of (a) L. lactis NZ9000 (WT) and its menaquinone deficient strains (∆menA, ∆menB, ∆menE, ∆menG), (b) L. lactis ∆menA and its engineered strains (lbmenA, lpmenA1, lpmenA2, lpmenA3), (c) L. lactis ∆menB, L. lactis ∆menE and its engineered strains (lbmenB, lbmenE) and (d) L. lactis ∆menG and its engineered strains (lbmenG, lpmenG1, lpmenG2). 300 mL of GM17 medium supplemented with heme (2 µg/mL) was used, 1 ng/mL of nisin was added as inducer at OD600 = 0.4, Figure S5: Growth profile (A) and pH analysis (B) of L. lactis NZ9000 (WT) and engineered deficient strains complemented by homologous genes (llmenA, llmenB, llmenE, llmenG). 300 mL of GM17 medium supplemented with heme (2 µg/mL) were used, 1 ng/mL of nisin was added as an inducer at OD600 = 0.4, Table S1: The Cre-lox system plasmids and knockout plasmid constructs, Table S2: Sequence of oligonucleotide primers, Table S3: Primers used for the functional verification of menaquinone biosynthesis genes study, Table S4: Plasmids and menaquinone expression plasmid constructs, Table S5: Lactococcus lactis strains with menaquinone expression plasmid, Table S6: Primers used for the reconstitution of menaquinone biosynthesis pathway in Lactipl. plantarum and Lent. buchneri strains.
