# Supplementary material for: Analysis and Reconstitution of the Menaquinone Biosynthesis Pathway in Lactiplantibacillus plantarum and Lentilactibacillus buchneri
Source: Microorganisms. 2021 Jul 9;9(7):1476. doi: 10.3390/microorganisms9071476 (PMC8303990; doi:10.3390/microorganisms9071476)
Supplement: Supplementary file 1 [file microorganisms-09-01476-s001.zip › microorganisms-1284923-supplementary.pdf]

**Supplementary Material**

**Table S1.** The Cre-*lox* system plasmids and knockout plasmid constructs

| Plasmids | Relevant features | References |
|---|---|---|
| pNZ5319 | $Cm^r$, $Ery^r$, containing *lox66*-P32-*cm*-*lox71* fragment | (Lambert et al., 2007) |
| pNZ5348 | $Ery^r$, Cre-recombinase expression | (Lambert et al., 2007) |
| pNZ5319-Δ*menA* | $Cm^r$, $Ery^r$; pNZ5319 derivative containing homologous regions upstream and downstream of NZ9000 *menA* | This study |
| pNZ5319-Δ*menB* | $Cm^r$, $Ery^r$; pNZ5319 derivative containing homologous regions upstream and downstream of NZ9000 *menB* | This study |
| pNZ5319-Δ*menE* | $Cm^r$, $Ery^r$; pNZ5319 derivative containing homologous regions upstream and downstream of NZ9000 *menE* | This study |
| pNZ5319-Δ*menG* | $Cm^r$, $Ery^r$; pNZ5319 derivative containing homologous regions upstream and downstream of NZ9000 *menG* | This study |

**Table S2.** Sequence of oligonucleotide primers

| Primer | Nucleotides |
|---|---|
| up A F *Xho*I | CGTCTCGAGATTGTTCCTAATGGCTCAAAA |
| up A R *Swa*I | TGCATTTAAATTTCCTCTTTTAATGTGATTTATCAA |
| down A F *Sac*I | TCCGAGCTCATAAAAAACTCCAATTAATTAA |
| down A R *Bgl*II | CGAAGATCTTTTTATTTAAAGCTTATAACTG |
| up B F *Xho*I | GATCTCGAGTCGGCTCCCGTTGAAAAA |
| up B R *Swa*I | GAAATTTAAATGATTTCTCCTTTAAGATGTGAGGG |
| down B F *Ban*II | TCCGRGCYCATAAAGCGTCATTTTGGCGC |
| down B R *Bgl*II | CGAAGATCTATGTTTATTTTTCCAGAATTGACCACTTC |
| up E F *Xho*I | CCGCTCGAGACTGGGTTGCCCTTA |
| up E R *Swa*I | CGCATCATTTAAATAGCGCCAAAATGACG |
| down E F *Ban*II | TCCGAGCTCAAGGGCCTTTCCGGAT |
| down E R *Bgl*II | CGAAGATCTACCGGCCATAGCATTTCC |
| up G F *Xho*I | GATCTCGAGCCTGACGTCGAGTCTG |
| up G R *Swa*I | GTCATTTAAATACTGGTCACAAGGTCAGC |
| down G F *Sac*I | GCCGAGCTCAGTTTTGAACGCTTGGATAG |
| down G R *Bam*HI | TACGGATCCTCGAGCACGCTTTCTTTTAT |
| lox F | AAATCTACCGTTCGTATAATGTATGC |
| lox R | CTCATGCCCGGGCTGTACCG |
| menA F | GTTTCTGCTGGGCCTTTCGTAC |

| | |
|---|---|
| men A R | GACAATGGTTTTGGGCCTCCTG |
| men B F | TCGCAATGCTTTTCGTCCAA |
| men B R | TCCCGCAAATTGTTGCAGTC |
| men E F | ATGAAATGGTTAAAAAAACAGGCGG |
| men E R | TCATGCTTTGAGCTCTTT |
| men G F | AACGAAGAACGTGTGCAAGA |
| men G R | AAGCGTCTTGGCATCTGGAA |
| Ery F | CGATACCGTTTACGAAAT |
| Ery R | CTTGCTCATAAGTAACGG |

*Underlined sequences indicate the restriction sites.

**Table S3.** Primers used for the functional verification of menaquinone biosynthesis genes study

| Primers | Nucleotides |
|---|---|
| LpA1 F | GGAGTACTTTGAAACCAAAAGTGTTTCTTGAA |
| LpA1 R | CTAGAGCTCCTAGTTAAAAATCAGTCCTAAGCC |
| LpA2 F | GGAGTACTTTGTTGACGAACGAACCAAC |
| LpA2 R | CTAGAGCTCTTAAAAGAAAATACCCAGGATCAGTC |
| LpA3 F | GGAGTACTATGTTTAAAAAATGGCTAACTTG |
| LpA3 R | CTAGAGCTCTTACAACCAAGTCCCTAAAACAAA |
| LpG1 F | GGAGTACTATGACAGCTTTAGAAAGAAGTTG |
| LpG1 R | TACGAGCTCTCAATCACGACGAGCCA |
| LpG2 F | GGAGTACTATGGCAAATCGTTATTTAC |
| LpG2 R | CTAGAGCTCTTACTTGGCCTCCTTAG |
| LbA F | GGAGTACTATGAGTTTATCAACTTTTTCCCAG |
| LbA R | CTAGAGCTCCTAGTGGGCAATCAGGG |
| LbG F | GGAGTACTATGACGCTGACGAACAA |
| LbG R | CTAGAGCTCTCAGGGCTTCATTCCCCA |
| LbB F | GGAGTACTATGACTTCAGTTAAATGGGAATC |
| LbB R | CTAGAGCTCCTATGGGAACTTAGGAAATTG |
| LbE F | GGAGTACTATGAAAGTGGATAATTGGATTTTAA |
| LbE R | CTAGAGCTCTTATAACAACTGTTTCAGCTTGAATC |
| LLA F | GGAGTACTATGAATTTTAAAACATTCGCT |

| | |
|---|---|
| LLA R | GGCGAGCTCTTAAAATCTAATCAAACTAATAAAGA |
| LLB F | GGAGTACTATGTCAAAATTTAACTGGGTTGC |
| LLB R | GGCGAGCTCTTATGGGAATTTTGGAAATTGGTC |
| LLE F | GGAGTACTATGAAATGGTTAAAAAAACAGGCG |
| LLE R | AACGGYRCCTCATGCTTTGAGCTCTTTTCTTAA |
| LLG F | GGAGTACTATGACTAAAGTAAACGAAGAACGT |
| LLG R | GGCGAGCTCTTACTTTTTACCAATACGAATATTTGATTT |
| pnz8150 F | GCATAATAAACGGCTCTGAT |
| pnz8150 R | CAGCAATATCAGTAATTGCTTTATC |

*Underlined sequences indicate the restriction sites.

**Table S4.** Plasmids and menaquinone expression plasmid constructs

| Plasmids | Relevant features | References |
|---|---|---|
| pNZ8150 | $Cm^r$, nisA promoter, expression vector | (Mierau and Kleerebezem, 2005) |
| pNZ8150-*lpmenA1* | $Cm^r$; nisA promoter, *lpmenA1* gene cloned to the MCS | This study |
| pNZ8150-*lpmenA2* | $Cm^r$; nisA promoter, *lpmenA2* gene cloned to the MCS | This study |
| pNZ8150-*lpmenA3* | $Cm^r$; nisA promoter, *lpmenA3* gene cloned to the MCS | This study |
| pNZ8150-*lpmenG1* | $Cm^r$; nisA promoter, *lpmenG1* gene cloned to the MCS | This study |
| pNZ8150-*lpmenG2* | $Cm^r$; nisA promoter, *lpmenG2* gene cloned to the MCS | This study |
| pNZ8150-*lbmenA* | $Cm^r$; nisA promoter, *lbmenA* gene cloned to the MCS | This study |
| pNZ8150-*lbmenB* | $Cm^r$; nisA promoter, *lbmenB* gene cloned to the MCS | This study |
| pNZ8150-*lbmenE* | $Cm^r$; nisA promoter, *lbmenE* gene cloned to the MCS | This study |
| pNZ8150-*lbmenG* | $Cm^r$; nisA promoter, *lbmenG* gene cloned to the MCS | This study |
| pNZ8150-*llmenA* | $Cm^r$; nisA promoter, *llmenA* gene cloned to the MCS | This study |
| pNZ8150-*llmenB* | $Cm^r$; nisA promoter, *llmenB* gene cloned to the MCS | This study |
| pNZ8150-*llmenE* | $Cm^r$; nisA promoter, *llmenE* gene cloned to the MCS | This study |
| pNZ8150-*llmenG* | $Cm^r$; nisA promoter, *llmenG* gene cloned to the MCS | This study |

**Table S5.** *Lactococcus lactis* strains with menaquinone expression plasmid

| Strains | Relevant features | References |
|---|---|---|
| *Lactococcus lactis* (ll) | | |
| *lpmenA1* | NZ9000-Δ*menA* carrying pNZ8150-*lpmenA1* | This study |
| *lpmenA2* | NZ9000-Δ*menA* carrying pNZ8150-*lpmenA2* | This study |
| *lpmenA3* | NZ9000-Δ*menA* carrying pNZ8150-*lpmenA3* | This study |
| *lpmenG1* | NZ9000-Δ*menG* carrying pNZ8150-*lpmenG1* | This study |
| *lpmenG2* | NZ9000-Δ*menG* carrying pNZ8150-*lpmenG2* | This study |
| *lbmenA* | NZ9000-Δ*menA* carrying pNZ8150-*lbmenA* | This study |
| *lbmenB* | NZ9000-Δ*menB* carrying pNZ8150-*lbmenB* | This study |
| *lbmenE* | NZ9000-Δ*menE* carrying pNZ8150-*lbmenE* | This study |
| *lbmenG* | NZ9000-Δ*menG* carrying pNZ8150-*lbmenG* | This study |
| *llmenA* | NZ9000-Δ*menA* carrying pNZ8150-*llmenA* | This study |
| *llmenB* | NZ9000-Δ*menB* carrying pNZ8150-*llmenB* | This study |
| *llmenE* | NZ9000-Δ*menE* carrying pNZ8150-*llmenE* | This study |
| *llmenG* | NZ9000-Δ*menG* carrying pNZ8150-*llmenG* | This study |

**Table S6.** Primers used for the reconstitution of menaquinone biosynthesis pathway in *Lactipl. plantarum* and *Lent. buchneri* strains

| Primers | Nucleotides |
|---|---|
| Psip 409 BsaIKO F | CACGTTACTAAAGGAAATGGAGACCGGGGT |
| Psip409 BsaIKO R | CGGTCGCCATTCCCTTTAGTAACGTGTAACTTTCCAAAT |
| Psip409 F BsaI | AAGGGTCTCATGCGTCTAGACTCGAGGAATT |
| Psip409 R BsaI | CATGGTCTCCGATCGCTAAAATCTCCTTGTAATA |
| **Golden Gate Assemble for *Lactipl. plantarum* WCFS1 menaquinone expression vector construction** | |
| Psip409 backbone F BsaI | AAGGGTCTCATGCGTCTAGACTCGAGGAATT |
| Psip409 backbone R BsaI | CATGGTCTCCCGCCGCTAAAATCTCCTTGTAATA |
| GO menF Lp F | TATGGTCTCAGCGGATTACAAGGAGATTTTAGCCATGAAATATATAAAAAACGATTTAATATTAA |
| GO menF Lp R | CGCGGTCTCATCTTTCATAAGGCTTCTAAAA |
| GO menD Lp F | TATGGTCTCAAGATATTACAAGGAGATTTTAGCCATGACCAATGAATATTTAGCTCC |
| GO menD Lp R | CGCGGTCTCCCTGATCAATTTTCATAAGCAGTATATTTTTTAT |
| GO menH Lp F | TATGGTCTCATCAGATTACAAGGAGATTTTAGCCATGAAAATTGATAAAAAAATAATGACGA |
| GO menH Lp R | CGCGGTCTCGTAAGCCTAAGCCAAAAATTCCTC |
| PsppQ LP F | TATGGTCTCGCTTAGGAGATCTACCGGTTTAATTTGAAA |
| PsspQ LP R | CGCGGTCTCGGCTCCGCGGCTAAAATCTCCTTGTAATAGT |

| | |
|---|---|
| GO menB Lp F | TATGGTCTCGGAGCATTACAAGGAGATTTTAGCCATGTCAAAATTTAACTGGGTTG |
| GO menB Lp R | CGCGGTCTCGAACCTTATGGGAATTTTGGAAATTGG |
| GO menE Lp F | TATGGTCTCAGGTTATTACAAGGAGATTTTAGCCATGAAATGGTTAAAAAAACAGGC |
| GO menE Lp R | CGCGGTCTCTGTAGTCATGCTTTGAGCTCTTT |
| GO menC LP F | TATGGTCTCACTACATTACAAGGAGATTTTAGCCATGAAAATTGAAAAAATCACAATGT |
| GO menC Lp R | CGCGGTCTCTCGCATCATTTCAAGGAGGTCAAGC |

*Underlined sequences indicate the restriction sites

Ribosome binding site are highlighted in grey

Colored nucleotides represent the overhangs for Golden Gate Assembly and the same color showed the complementary bases

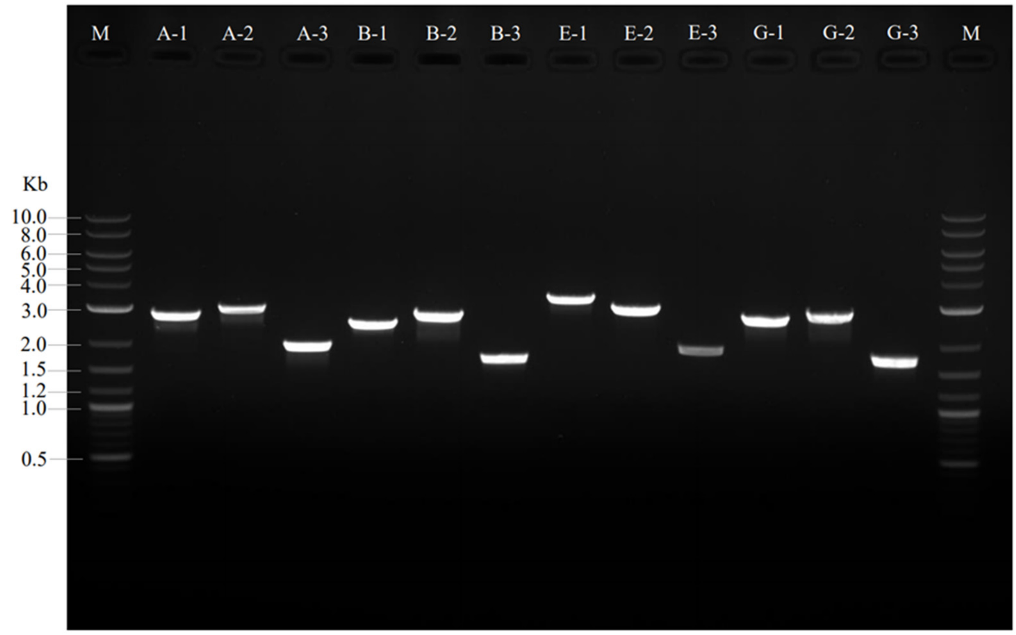


**Figure S1.** Diagnostic PCR of *L. lactis* NZ9000 *menA* (A-1), its gene replacement mutant *menA*:: *lox66*-P32-*cm-lox71* (A-2), *ΔmenA* (A-3); *menB* (B-1), *menB*:: *lox66*-P32-*cm-lox71* (A-2), *ΔmenB* (B-3); *menE* (E-1), *menE*:: *lox66*-P32-*cm-lox71* (E-2), *ΔmenE* (E-3); *menG* (G-1), *menG*:: *lox66*-P32-*cm-lox71* (A-2), *ΔmenG* (G-3); M is a 2 log DNA ladder

.

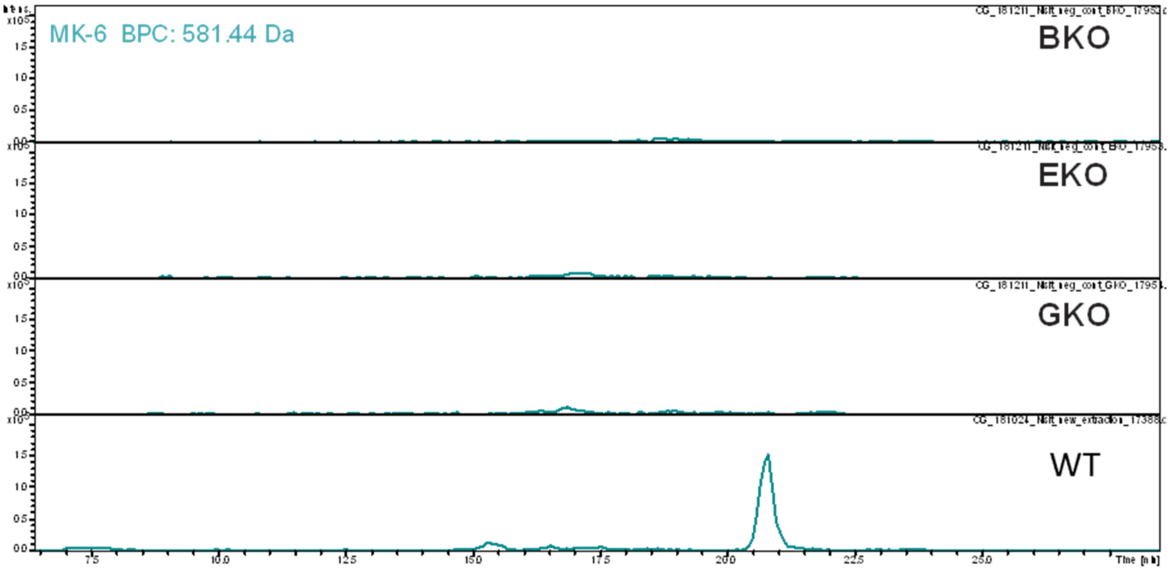


**Figure S2.** Base peak chromatogram of menaquinone extracts from *L. lactis* NZ9000 (WT) and its menaquinone deficient strains Δ*menB* (BKO), Δ*menE* (EKO), Δ*menG* (GKO) for MK-6.

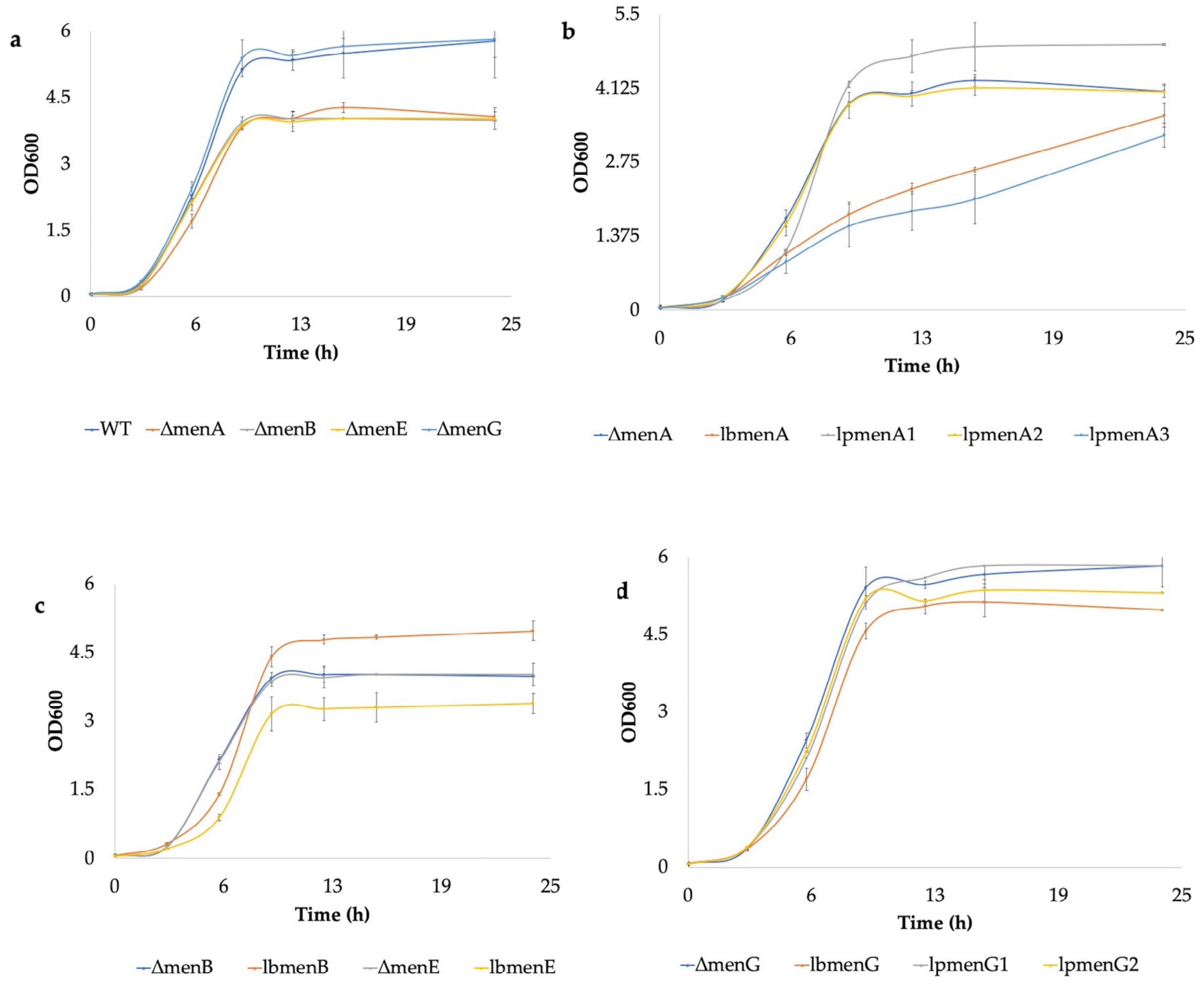


**Figure S3.** Growth profile analysis of (a) *L. lactis* NZ9000 (WT) and the knockout strains (Δ*menA*, Δ*menB*, Δ*menE*, Δ*menG*), (b) *L. lactis* Δ*menA* and its engineered derivatives carrying *lbmenA*, *lpmenA1*, *lpmenA2* and *lpmenA3*), (c) *L. lactis* Δ*menB*, *L. lactis* Δ*menE* and the engineered derivatives (carrying *lbmenB*, *lbmenE*) and (d) *L. lactis* Δ*menG* and its engineered derivatives carrying *lbmenG*, *lpmenG1* and *lpmenG2*. 300 mL of GM17 medium supplemented with heme (2μg/mL) were used, 1 ng/mL of nisin was added as an inducer at OD$_{600}$ = 0.4.

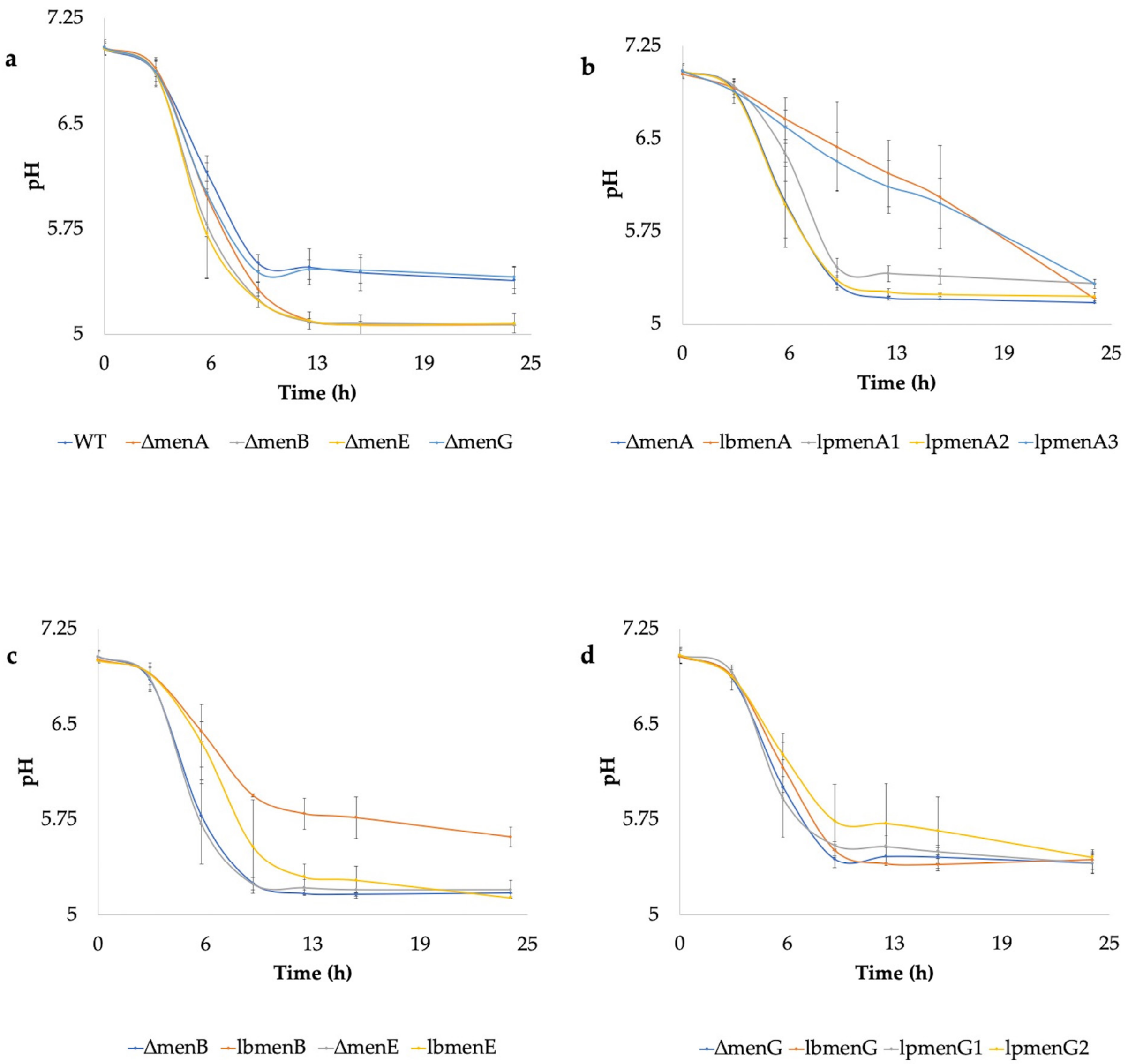


**Figure S4.** pH profile of (a) *L. lactis* NZ9000 (WT) and its menaquinone deficient strains (Δ*menA*, Δ*menB*, Δ*menE*, Δ*menG*), (b) *L. lactis* Δ*menA* and its engineered strains (lbmenA, lpmenA1, lpmenA2, lpmenA3), (c) *L. lactis* Δ*menB*, *L. lactis* Δ*menE* and its engineered strains (lbmenB, lbmenE) and (d) *L. lactis* Δ*menG* and its engineered strains (lbmenG, lpmenG1, lpmenG2). 300 mL of GM17 medium supplemented with heme (2μg/mL) was used, 1 ng/mL of nisin was added as inducer at $OD_{600}$ = 0.4.

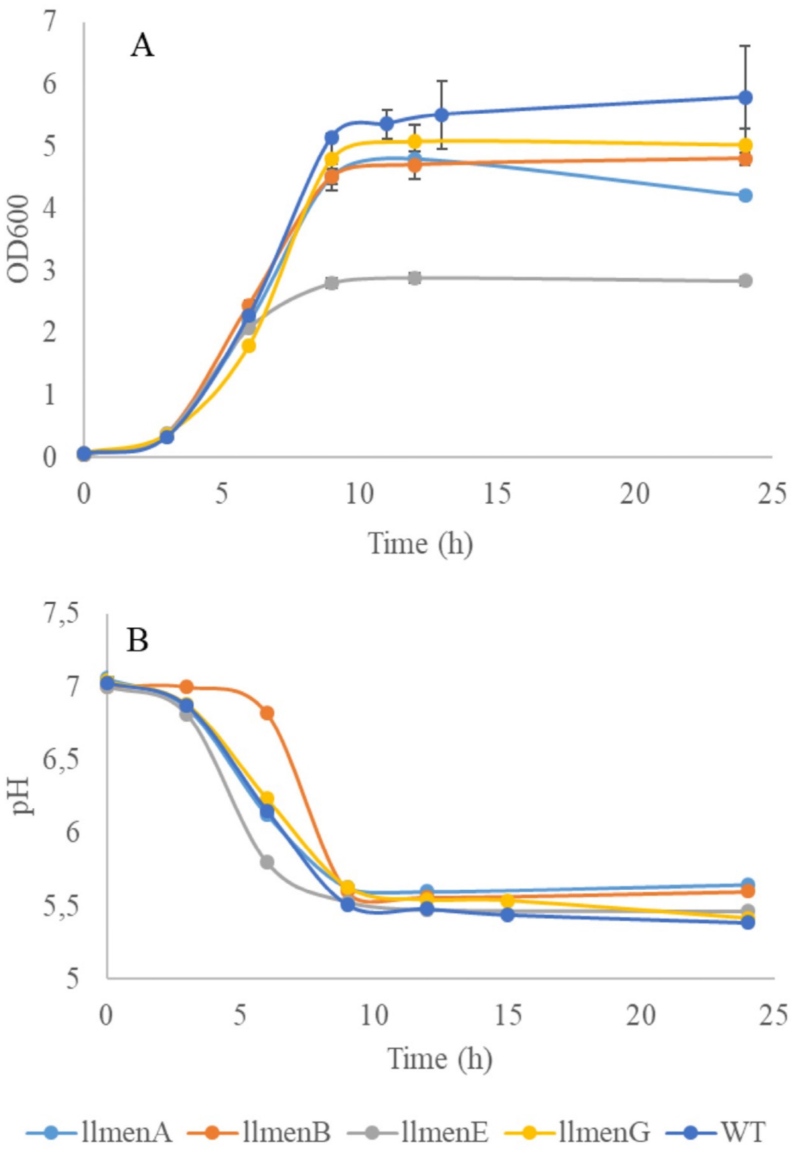


**Figure S5.** Growth profile (A) and pH analysis (B) of *L. lactis* NZ9000 (WT) and engineered deficient strains complemented by homologous genes (*llmenA*, *llmenB*, *llmenE*, *llmenG)*. 300 mL of GM17 medium supplemented with heme (2µg/mL) were used, 1 ng/mL of nisin was added as an inducer at $OD_{600}$ = 0.4.
